# Epicutaneous Immunization with Type II Collagen Inhibits both Onset and Progression of Chronic Collagen-Induced Arthritis

**DOI:** 10.1371/journal.pone.0000387

**Published:** 2007-04-18

**Authors:** Jessica Strid, Lee Aun Tan, Stephan Strobel, Marco Londei, Robin Callard

**Affiliations:** 1 Immunobiology Unit, Institute of Child Health, University College London, London, United Kingdom; 2 Autoimmunity and Gastroenterology Unit, Institute of Child Health, University College London, London, United Kingdom; 3 Peninsula Postgraduate Health Institute, Plymouth, United Kingdom; Centre de Recherche Public-Santé, Luxembourg

## Abstract

Epicutaneous immunization is a potential non-invasive technique for antigen-specific immune-modulation. Topical application of protein antigens to barrier-disrupted skin induces potent antigen-specific immunity with a strong Th2-bias. In this study, we investigate whether the autoimmune inflammatory response of chronic collagen-induced arthritis (CCIA) in DBA/1-TCR-β Tg mice can be modified by epicutaneous immunization. We show that epicutaneous immunization with type II collagen (CII) inhibited development and progression of CCIA and, importantly, also ameliorated ongoing disease as indicated by clinical scores of disease severity, paw swelling and joints histology. Treated mice show reduced CII-driven T cell proliferation and IFN-γ production, as well as significantly lower levels of CII-specific IgG2a serum antibodies. In contrast, CII-driven IL-4 production and IgE antibody levels were increased consistent with skewing of the CII response from Th1 to Th2 in treated mice. IL-4 production in treated mice was inversely correlated with disease severity. Moreover, T cells from treated mice inhibited proliferation and IFN-γ production by T cells from CCIA mice, suggesting induction of regulatory T cells that actively inhibit effector responses in arthritic mice. The levels of CD4^+^CD25^+^ T cells were however not increased following epicutaneous CII treatment. Together, these results suggest that epicutaneous immunization may be used as an immune-modulating procedure to actively re-programme pathogenic Th1 responses, and could have potential as a novel specific and simple treatment for chronic autoimmune inflammatory diseases such as rheumatoid arthritis.

## Introduction

The route of antigen administration is a key factor in determining the nature of an immune response. The different responses obtained may enable alternative routes of antigen delivery to be used for generating therapeutic immune responses. In recent years, it has become clear that application of antigen onto bare skin induces potent systemic and mucosal immunity in an antigen-specific manner [1]–[3]. Several strategies to exploit the immune system of the skin for needle-free vaccine delivery are currently being developed [4], [5]. These new strategies rely however on the use of strong non-specific adjuvants for induction of good immune responses, some of which are toxic and generally not acceptable for human immunization. We have recently shown that a natural adjuvant effect can be achieved by simply disrupting the stratum corneum of the epidermis prior to topical antigen application [1]. This activates the resident Langerhans cells and negates the need for coapplication of potentially noxious adjuvants. We have shown previously that epicutaneous immunization on barrier-disrupted skin induces potent and strongly Th2-biased immunity [1], [6]. This is consistent with other studies showing that the epicutaneous microenvironment appears to naturally favour the induction of Th2 and generally anti-inflammatory responses [2], [3], [7]. Furthermore, we have shown that the Th2 response induced by epicutaneous immunization exerts a dominant effect over CFA-induced Th1 responses by interfering with the development of systemic antigen-specific Th1 responses and skewing established Th1 responses towards Th2 [6]. These observations suggest that this route of antigen delivery could be of potential therapeutic benefit in Th1-type autoimmune diseases such as rheumatoid arthritis (RA).

RA is an autoimmune disease characterized by chronic inflammation, cyclic progressive evolution and ensuing destruction of cartilage and bone, leading to severe disability. The disease has a prevalence of 1%, making RA one of the most common chronic inflammatory diseases [8], [9]. In spite of intense research, many of the principles behind the pathogenic mechanisms of RA still remain to be elucidated. However, cumulative evidence suggests that CD4^+^ T lymphocytes predominantly expressing a Th1 cytokine pattern drives the pathology [10], [11]. Joint-specific autoantigens, such as type II collagen (CII) are thought to play a key role by instigating T cell mediated immune responses [12] with autoantibodies subsequently also being involved [8]. Several therapeutic strategies are available for RA and continuous improvement and refinement of these therapies have had a profound impact on progression of disease and quality of life for RA patients [13]. None of the currently used therapies are, however, disease- or antigen-specific and there is therefore a need for developing new disease-specific therapies which may benefit RA patients.

DBA/1-TCR-β Tg mice develop a chronic relapsing polyarthritis with an incidence approaching 100% after immunization with CII in CFA. This model of chronic collagen-induced arthritis (CCIA) is dependent on CD4^+^ Th1 cells and shares key characteristics with human RA [12]. In this study, we use the CCIA model to investigate the potential of epicutaneous immunization to provide a means of modulating RA. We report, that epicutaneous immunization with CII protects against the development of CCIA and importantly is also effective in ameliorating disease when given after induction of CCIA. Epicutaneous immunization, whether prior or post induction of disease, inhibits pathogenic CII-driven Th1 responses and enhances anti-inflammatory Th2 responses. These results suggest, that this route of active autoantigen immunization may provide an alternative or complimentary therapeutic strategy for autoimmune diseases such as RA.

## Results

### Epicutaneous immunization with CII inhibits development of CCIA

**Figure 1:**
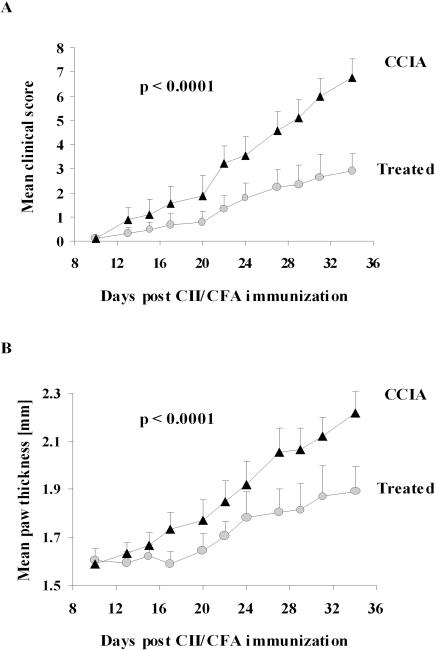
Epicutaneous immunization prevents evolution of arthritis. DBA/1-TCR-β Tg mice were epicutaneously immunized with CII 3 weeks prior to induction of disease by injection of CII in CFA. Mice were evaluated and clinical score (A) and hind paw thickness (B) were assessed every second day. Data represents the mean+1 SEM (n = 9). The epicutaneous immunization had a highly significant effect on disease severity as analysed by two-way ANOVA. The entire *in vivo* experiment was repeated twice with very similar results. (•) = treated with epicutaneous CII, (▴) = sham-treated CCIA controls.

Topical application of protein antigen to skin from which the stratum corneum has been removed with adhesive tape gives rise to potent systemic immune responses [1]. Immunity initiated via such epicutaneous immunization is strongly Th2-biased with high levels of antigen-specific IgG1 and IgE and enhanced production of IL-4 but with little or no IgG2a and IFN-γ [1]. The Th2 responses obtained by this route are dominant over Th1 responses induced by antigen in CFA [6], suggesting that epicutaneous immunization with an RA autoantigen could modulate disease in the CCIA model. To examine this possibility, DBA/1-TCR-β Tg mice were immunized epicutaneously with CII three weeks prior to induction of arthritis. Development of clinical disease was closely monitored for five weeks following arthritis induction (Figure 1). A highly significant decrease in disease severity as well as a delay in the onset and an overall lower incidence of disease was observed in treated mice compared to sham treated CCIA diseased controls. The treated mice that did show disease developed a milder arthritis (p<0.0001) (Figure 1A) with reduced paw swelling (p<0.0001) (Figure 1B) and with fewer joints involved (p<0.0001) (data not shown). These results demonstrated a highly significant effect of epicutaneous immunization on both the incidence and evolution of clinical arthritis.

### Histological features

**Figure 2:**
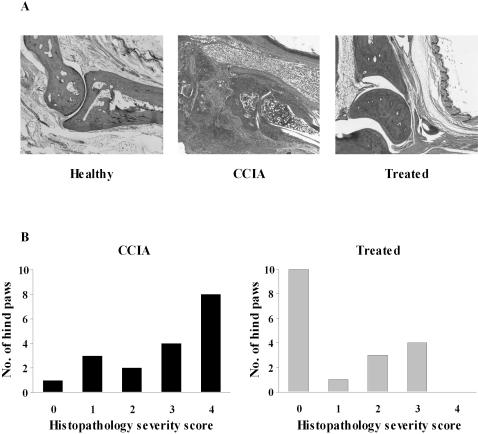
Histopathological damage of joints is reduced following epicutaneous immunization. Hind paws from healthy mice, CCIA control mice and epicutaneously treated mice were sectioned and stained with H&E (A). One representative example is shown. Note that skin and muscle remain unaffected in CCIA mice. Histological sections were evaluated and arbitrary scores assigned in 5 categories (4 being the most severe score) for extent of inflammatory infiltrate, cartilage and bone erosion. The number of affected hind paws assigned each severity score is shown (B). The mean histopathological severity score was significantly lower in treated mice, p<0.003 (n = 18). Healthy = un-CFA-immunized non-arthritic littermate, CCIA (▪) = sham-treated arthritic mouse, Treated (▪) = epicutaneously immunized mouse.

Hind paws were collected at the end of the experiment and joints evaluated histologically to determine whether the amelioration of disease in the treated mice correlated with reduced joint damage (Figure 2A). Sections of hind paws from healthy control animals showed open joint spaces free of cells, bones were closely apposed with smooth surfaces of bone and cartilage and no inflammatory cellular infiltrates were visible. Diseased CCIA mice had an extensive inflammatory infiltrate in the joint spaces, contours of bone and cartilage were irregular and in most cases there was severe pannus invasion. Pannus extended over the articular cartilage, eroded the junction of the cartilage and bone and in many cases expanded into the bone marrow space. In these severely diseased CCIA mice there was a complete loss of bone architecture with a collapse of the joint and extensive bone erosion. In sharp contrast, most of the joint sections from mice treated by prior epicutaneous immunization with CII showed no signs of inflammation, synovitis or any affect on cartilage or bone. Surfaces of bone and cartilage were in most cases smooth and bone architecture appeared normal. Some treated mice did show joint damage with some inflammatory infiltrate and erosion of cartilage, but these cases were much milder than the majority of CCIA control mice and bone architecture was never completely lost (Figure 2A). To obtain a quantitative measure of the effect of treatment on histopathology, sections of both hind paws of all mice were scored for the severity of joint damage, extent of inflammation and bone erosion using an arbitrary scale by independent investigators ‘blinded’ to mouse identity. The analysis showed that the histopathology of joints from treated mice were significantly milder than in sham-treated control mice (p<0.003). 10/18 joints from treated mice showed no signs of pathology whereas the rest showed varying degrees of inflammatory infiltrate and cartilage erosion. None however showed severe bone destruction with complete loss of bone architecture (Figure 2B). These results illustrate the potent suppressive effect of epicutaneous immunization on the inflammatory processes leading to joint destruction.

### Epicutaneous immunization inhibits CII-driven proliferation and skews the pathogenic CII Th1 response to a Th2 response

**Figure 3:**
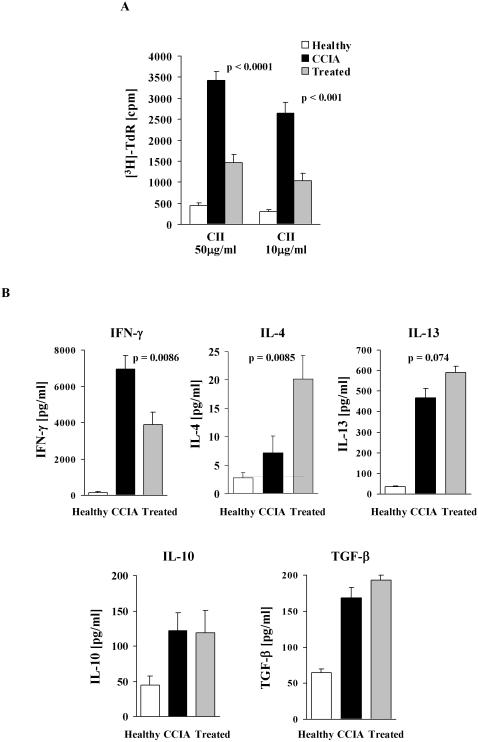
Epicutaneous immunization inhibits CII-specific proliferation, reduces Th1 and enhances Th2-type cytokines. Splenocytes were collected from each mouse and cultured for 90 h with CII or control antigen. Proliferation was determined by [^3^H]-thymidine incorporation (A) and cytokine production by ELISA (B). Results of proliferative experiments are expressed as mean cpm+1 SEM (n = 9). Background proliferation when no antigen was present has been subtracted. No proliferation was observed to control antigen but the two groups responded with similar [cpm] when stimulated with ConA. Production of IFN-γ, IL-4, IL-13, IL-10 and TGF-β is expressed as mean pg/ml+1 SEM (n = 9). No cytokines were produced in response to a control antigen or when cells were not stimulated. Dashed horizontal lines (where visible) represents limit of detection. (□) = un-immunized healthy controls, (▪) = treated with epicutaneous CII, (▪) = sham-treated CCIA controls.

**Figure 4:**
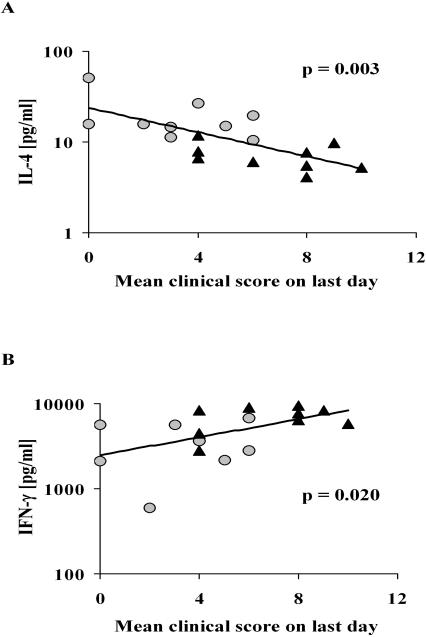
CII-driven production of IL-4 correlates inversely with severity of arthritis. The linear correlation between Th2/Th1 cytokine production and severity of arthritis following epicutaneous immunization was analysed using linear regression. Production of IL-4 was negatively correlated to the clinical score (A) while production of IFN-γ was positively correlated to clinical score (B). Each point represents one mouse. (•) = treated with epicutaneous CII, (▴) = sham-treated CCIA controls.

Our previous studies have shown that the epicutaneous immunization procedure used here induces a potent Th2 response that is able to override an ongoing Th1 response [1], [6], suggesting that this may be the mechanism responsible for the effect on clinical CCIA shown above. To examine this hypothesis, splenocytes were collected from all animals at the end of the experiment and CII-driven T cell proliferation as well as cytokine release were determined. In both the CCIA arthritic and treated groups, dose-dependent proliferation of splenic T cells was demonstrated to CII but not to control antigen, while T cells from healthy (un-immunized) mice only proliferated marginally above background when stimulated with CII (Figure 3A). The response of T cells from treated mice was however significantly reduced compared to T cells from sham-treated CCIA arthritic mice (Figure 3A). The CII-driven proliferation by T cells from treated mice was consistently about 60% lower than for T cells from CCIA mice, regardless of the CII concentration used. Cytokines produced by CII activated T cells from the experimental groups were also determined (Figure 3B). High levels of IFN-γ and low levels of IL-4 were secreted by splenocytes from CCIA diseased mice in response to CII stimulation. In contrast, IFN-γ production was significantly reduced in treated animals (p<0.01). This decrease in IFN-γ was mirrored by a concomitant and significant increase in IL-4 (p<0.01) and to a lesser extent IL-13 production (p = 0.07). The level of CII-driven IL-4 production in both groups of animals demonstrated a significant inverse linear correlation with disease severity (Figure 4A). Conversely, production of IFN-γ showed a positive linear correlation to the severity of arthritis, with treated animals producing the lowest amount of IFN-γ (Figure 4B). Similar levels of CII-driven IL-10 and TGF-β were induced in the two groups (Figure 3B). The levels of cytokines produced when splenocytes from healthy mice were stimulated with CII were below (IL-4) or only just over the detection limit of the assays (Figure 3B). No cytokines were produced to a control antigen or when cells were not stimulated (data not shown).

### CII-specific serum antibodies are reduced by epicutaneous immunization

**Figure 5:**
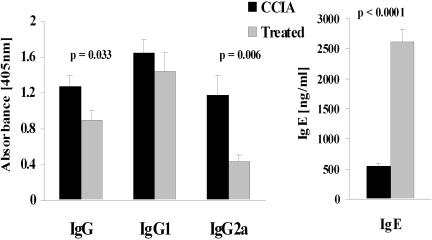
Epicutaneous immunization inhibits development of CII-specific IgG2a and IgG. CII-specific IgG, IgG1 and IgG2a as well as total IgE were measured in serum at the end of the experiment. Serum samples were diluted 1∶1500 for IgG, IgG1 and IgG2a and 1∶20 for IgE prior to analysis. Each bar represent mean antibody level+1 SEM (n = 9). (▪) = treated with epicutaneous CII, (▪) = sham-treated CCIA controls.

To determine whether epicutaneous immunization affected antibody responses to CII in CCIA diseased mice, levels of CII-specific IgG, IgG1 and IgG2a in sera were measured (Figure 5). The results showed that treated mice had greatly reduced levels of CII-specific IgG2a compared to CCIA arthritic mice (p<0.01) and this was mirrored by reduced CII-specific IgG (p<0.05) levels (Figure 5). The epicutaneous immunization had no significant effect on CII-specific IgG1. To further test whether the epicutaneous immunization had induced a shift in the nature of T cell help, levels of IgE were tested in the sera. Notably, the level of total IgE was greatly increased in the treated mice (p<0.0001). These results are consistent with the observed Th2 skewing of cytokine production and suggest that the epicutaneous immunization treatment restrained the widely recognized pathogenic Th1 CII specific response with a partial redirection towards a Th2 response.

### Lymphocytes from treated mice inhibit responses of T cells from CCIA arthritic mice

**Figure 6:**
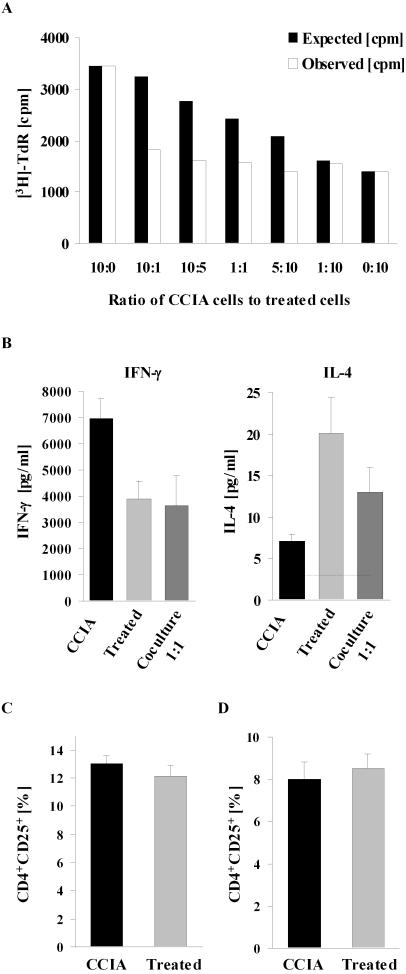
Lymphocytes from epicutaneously immunized mice inhibit proliferation and IFN-γ production by lymphocytes from CCIA mice. Splenocytes from a sham-treated CCIA control were cocultured with splenocytes from an epicutaneously treated mouse at varying ratios (A). Total number of cells in the culture was kept constant. Cocultures were stimulated with 50 µg CII/ml and CII-specific splenocyte proliferation was assayed as in Figure 3. Black bars demonstrate the expected proliferation [cpm] in the culture, calculated by the formula: [cpm] = a f+b(1−f), where a = [cpm] with 100% control cells, b = [cpm] with 100% treated cells, f = fraction of control cells in the culture. The observed [^3^H]-thymidine incorporation is shown in white bars and represents the mean of triplicate cultures. Similar results were obtained in four individual experiments. Production of IFN-γ and IL-4 in 1∶1 ratio cocultures of splenocytes from control and treated mice were assessed by ELISA and results are expressed as mean pg/ml+1 SEM (n = 4) (B). No cytokines were produced in response to a control antigen or when cells were not stimulated. The proportion of CD4^+^CD25^+^ T cells in freshly isolated splenocytes (C) from CCIA control or epicutaneously immunized mice and after 90 h *in vitro* stimulation with 50 µg CII/ml (D) were assessed by flow cytometry. Results are expressed as the mean [%] CD4^+^CD25^+^ T cells out of the total CD4^+^ population+1 SEM (n = 9). (▪) = treated with epicutaneous CII, (▪) = sham-treated CCIA controls.

Epicutaneous immunization inhibited T cell proliferation to CII in treated mice as shown in Figure 3. We therefore considered the possibility that epicutaneous immunization may generate T cells that can inhibit the effector function of T cells from mice with CCIA. To examine this possibility, T cells from CCIA and treated mice were cocultured at various cell ratios and the proliferative response to CII stimulation was measured. The results showed that CII-driven proliferation by splenocytes from CCIA mice was inhibited when cocultured with splenocytes from treated mice (Figure 6A). Introducing as few as 10% of cells from treated mice into the cultures of cells from CCIA mice reduced the proliferative response to about half of that predicted from responses by cells from both groups of animals cultured alone. In addition, secretion of IFN-γ by cells from CCIA mice was completely inhibited by coculture with treated cells (p<0.05). Levels of IFN-γ were significantly below the levels expected from the cells cultured separately. Simultaneously, IL-4 was increased (p<0.02) but only to the predicted level (Figure 6B). No inhibitory effect was observed on proliferation or cytokine secretion when cells from two CCIA control mice were cocultured (data not shown). Nor was any inhibitory effect observed when the cells were stimulated with the mitogen Concanavalin A (ConA) (not shown), suggesting that inhibition was specific for the CII response.

There is good evidence that the CD4^+^CD25^+^ T cell subset (natural T regulatory cells) can confer protection in a variety of autoimmune disease models [14]–[16]. We therefore analysed whether a difference in the relative proportion of CD4^+^CD25^+^ T cells could be responsible for the reduced proliferation and inhibitory effect of T cells from epicutaneously treated mice. Splenocytes were stained for CD4^+^ and CD25^+^ T cells and analysed by flow cytometry. There were no differences in the proportion of CD4^+^CD25^+^ T cells from CCIA diseased mice and treated mice in either freshly isolated splenocytes (Figure 6C) or following 90 h re-stimulation with CII (Figure 6D). In previous experiments we had additionally demonstrated that there was no difference in the expression level of FoxP3 or Glucocorticoid-induced TNF receptor family related protein (GITR) between diseased CCIA and healthy un-immunized mice as assessed by flow cytometry (data not shown), therefore these markers of (natural) regulatory T cell function were not further evaluated in the experiments shown here.

### Treatment by epicutaneous immunization after induction of CCIA ameliorates arthritis

**Figure 7:**
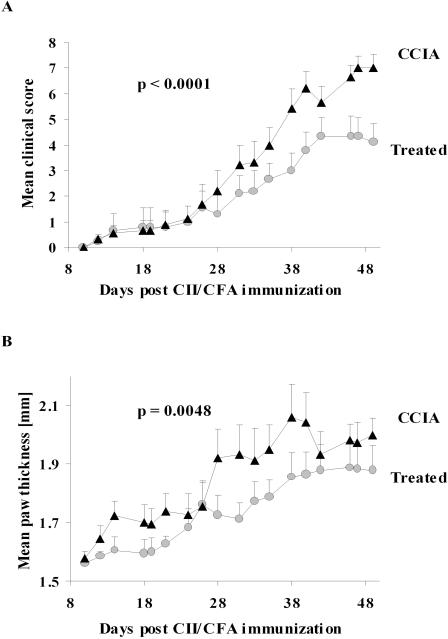
Treatment with epicutaneous immunization ameliorate ongoing arthritis. Arthritis were induced in Tg mice by injection of CII in CFA and 1 week later mice were treated with epicutaneous CII. Mice were evaluated and clinical score (A) and hind paw thickness (B) were assessed every second day for 49 days. Data represents the mean+1 SEM (n = 9). Treatment by epicutaneous immunization had a significant effect on disease severity as analysed by two-way ANOVA. (•) = treated with epicutaneous CII, (▴) = sham-treated CCIA controls.

The results so far show that prior epicutaneous immunization with CII profoundly inhibits CCIA induced three weeks later with CII in CFA. In order to investigate whether this form of treatment can also limit the progression of CCIA after induction of disease, DBA/1-TCR-β Tg mice were subjected to epicutaneous CII immunization one week after induction of disease by intradermal injection of CII in CFA. In these experiments there were no difference in the time of disease onset in the two groups but the progression and overall clinical severity of CCIA was significantly reduced in the group treated by epicutaneous CII immunization (p<0.0001) (Figure 7A). The significant reduction in arthritic severity was also reflected in reduced paw swelling (p<0.005) (Figure 7B) and the involvement of fewer joints (p<0.05) (not shown) compared to sham-treated CCIA controls.

**Figure 8:**
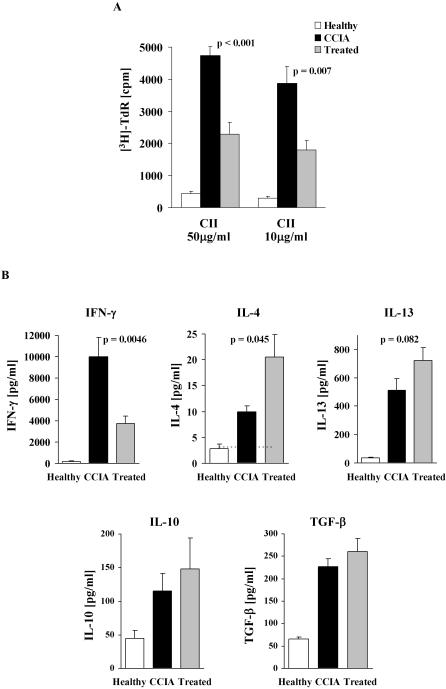
Treatment with epicutaneous immunization reduces CII-driven proliferation and IFN-γ production while IL-4 production is enhanced. Splenocytes were collected from all mice on day 49 after induction of disease and cultured with CII or control antigen for 90 h. CII-driven proliferation (A) and production of IFN-γ, IL-4, IL-13, IL-10 and TGF-β (B) were assayed as described in Figure 3. Results are expressed as mean+1 SEM (n = 9). (□) = un-immunized healthy controls, (▪) = treated with epicutaneous CII, (▪) = sham-treated CCIA controls.

Splenocytes collected from mice treated by epicutaneous CII after induction of CCIA also exhibited reduced dose-dependent T cell proliferation to CII stimulation compared to T cells from sham-treated CCIA mice (Figure 8A). In addition, the production of pathogenic IFN-γ in response to CII stimulation was significantly inhibited in treated mice (p<0.005) (Figure 8B) whereas IL-4 was significantly increased (p<0.05). The production of IL-13 was also consistently increased in treated mice, although this did not reach statistical significance. There was no significant difference in the CII-driven production of IL-10 or TGF-β between treated and non-treated mice (Figure 8B). Splenic T cells from healthy control mice showed only marginal T cell proliferation and cytokine production in response to CII stimulation (Figure 8).

**Figure 9:**
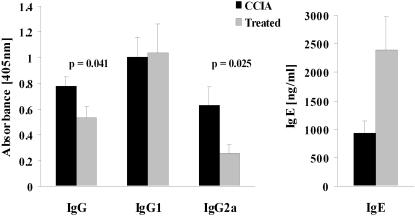
Treatment with epicutaneous immunization reduces levels of CII-specific IgG2a and IgG. CII-specific IgG, IgG1 and IgG2a as well as total IgE were measured in serum at the end of the experiment. Serum samples were diluted 1∶1500 for IgG, IgG1 and IgG2a and 1∶20 for IgE prior to analysis. Each bar represent mean antibody level+1 SEM (n = 9). (▪) = treated with epicutaneous CII, (▪) = sham-treated CCIA controls.

Serum levels of CII-specific IgG, IgG1 and IgG2a and total IgE were also measured 42 days after epicutaneous immunization (i.e. 49 days after induction of CCIA). Treated mice had significantly lower levels of anti-CII IgG2a (p<0.05) compared to CCIA controls (Figure 9). The total level of anti-CII IgG was also significantly reduced (p<0.05), presumably as a consequence of the reduced CII-specific IgG2a levels. Epicutaneous immunization had no effect on levels of CII-specific IgG1 but levels of total IgE were increased (p<0.05) (Figure 9). Treatment by epicutaneous CII immunization after induction of CCIA had thus caused a modulation of the anti-CII antibody response, resulting in a decrease in the ratio of IgG2a to IgG1 and a reduction in the levels of complement-fixing antibodies, consistent with a Th2 skew.

## Discussion

Epicutaneous immunization with protein or peptide antigens without adjuvants can induce a sustained Th2-type response that is able to skew an ongoing Th1 response towards Th2 as indicated by cytokine production and antibody response [1]–[3], [6]. In this study, we sought to utilise this route of immunization with CII to interfere with the development and evolution of CCIA disease in the DBA/1-TCR-β Tg mouse model of RA. RA has a number of systemic manifestations but it is the chronic inflammation in the joints that is the most prominent and consistently destructive feature of the disease [9]. There is compelling evidence that T cells are central in many autoimmune diseases, though complex interactions with other immuno competent cells are often required and necessary for the full profile of the disease. A number of studies have suggested that T cells in RA joints are predominantly of the Th1 phenotype [10], [13] and there is general consensus that a Th1 pro-inflammatory response initiates the whole pathogenic cascade. On that basis, it has been suggested that down-regulation of the Th1 response in RA could be beneficial for preventing the clinical progression of disease. We have shown here that epicutaneous immunization with CII significantly interferes with the development of CCIA. The incidence of disease was reduced in treated mice and when disease developed it showed a delayed onset and both the clinical phenotype and the pathological destruction of the bone were considerably milder than in control mice. Immunologically, epicutaneous immunization inhibited the development of CII-specific pathogenic Th1 responses by significantly reducing IFN-γ and IgG2a. Simultaneously, this route of CII administration induced active Th2 responses by enhancing CII-specific IL-4 and IL-13 and increasing levels of IgE. Importantly, a similar reduction in disease severity and intensity of CII-specific Th1 responses were observed when epicutaneous CII was administered after induction of disease. Again a significant skew from Th1 to Th2 immunity was apparent in cytokine production and IgG isotype. Moreover, disease severity was directly correlated with IFN-γ production and inversely correlated with levels of CII-induced IL-4 production. Together, these results suggests that epicutaneous immunization is effective in controlling arthritis by actively inhibiting autoantigenic and pathogenic Th1 responses and promoting specific anti-inflammatory Th2 responses. Even more effective inhibition of disease may be possible by repeated epicutaneous immunizations and further studies will address this possibility.

Recently, a subset of IL-17 producing T helper cells (Th17) distinct from Th1 and Th2 cells have been described [17], [18]. IL-17 appears to play a major role in several models of immune-mediated tissue injury, including organ-specific autoimmunity such as rheumatoid arthritis. Collagen-induced arthritis is suppressed in IL-17^-/-^ mice [19] and Th17 cells are involved in bone destruction and remodelling [20]. In this study we have focused on Th1 cells but further studies will identify whether the disease controlling effects of epicutaneous immunization on CCIA is additionally caused by an effect on Th17 cells.

Beneficial effects on the development of RA by inhibiting Th1 or enhancing Th2 responses have been demonstrated in previous studies. For example, neutralization of IFN-γ or IL-12 [21], [22] or administration of IL-4 [23] during the early phase of disease has been shown to exert a protective effect on the evolution of arthritis, although these strategies were less effective or ineffective if administered during established disease. In human RA, inhibition of pro-inflammatory cytokines such as IL-1β [24], [25] and notably TNF-α [13], [26] has proven effective in modifying disease and has profoundly improved the quality of life for some patients with severe RA. However, while it is possible to temporally down-regulate the Th1 response by passively blocking Th1-type cytokines or by administering Th2-type cytokines, the response reverts back to a Th1 response once treatment is stopped [23]. In this study, we sought to use a different strategy by inducing active Th2 responses through epicutaneous immunization for treatment of Th1-mediated arthritis. By actively immunizing through this route, self-sustainable clones of Th2 cells with a broader range of Th2 cytokine expression and subsequent continued Th2 help can be induced. Of major importance is the antigen-specificity of the active Th2 responses induced by epicutaneous immunization [6]. This should promote selective migration of CII-specific Th2 cells to the diseased tissue where they can exert their effect. At the site of disease activity they may also suppress pathogenic responses to other joint antigens by way of local bystander suppression, a phenomenon that has been demonstrated in several animal models of inflammatory autoimmune disease [27], [28]. Bystander suppression has also been reported *in vitro* in humans [29] but little is known about whether this occurs *in vivo* in humans or how it can be triggered. Another obvious advantage of inducing antigen-specific therapeutic responses is that Th1 responses to other antigens and pathogens such as mycobacterium should not be suppressed, which reduces the problematic augmented risk of infection that a non-specific approach carries [13].

Several mechanisms could be behind the inhibitory effect of epicutaneous immunization on pathogenic Th1 responses and disease severity. The observed overall switch from Th1 to Th2-type immunity may represent the *de novo* induction of a dominant antigen-specific Th2 population or may be due to an actual reversal in differentiation commitment of the CII-specific Th cells. Differentiation of Th1 and Th2 cells are strongly influenced by the presence of IFN-γ and IL-4 which control expression of the transcription factors T-bet [30] and GATA-3 [31]. T cell differentiation at a population level ultimately depends on the expression dynamics of T-bet and GATA-3, which are mutually exclusive [32]. It is theoretically possible to reverse Th cell commitment through manipulation of these transcription factors by IFN-γ and IL-4 [32]. Although this remains an exciting hypothetical possibility, it is not possible to determine from the experiments described here whether the switch to a Th2 dominant response following epicutaneous immunization represents a true re-programming of CII specific T cells or preferential expansion of Th2 T cells.

Another possible mechanism behind the distinct change in the Th cell profile after epicutaneous immunization is active suppression of the ongoing Th1 response allowing for emergence of Th2 cells. Our data suggests that epicutaneous immunization not only produces an immunological switch from Th1 to Th2 but may also trigger a suppressive/regulatory pathway. In cocultures, cells from mice treated by epicutaneously immunization were able to actively inhibit CII-specific proliferation and IFN-γ release by effector cells, suggesting an active regulatory mechanism. Adding as little as 10% of cells from treated mice to cultures of cells from diseased CCIA mice completely inhibited IFN-γ production, showing the dominant suppressive effect of the epicutaneously induced T cells. We did not however detect an increase in the proportion of CD4^+^CD25^+^ T cells in treated mice, which may suggest that the induced suppressive effect does not involve the naturally occurring CD4^+^CD25^+^ regulatory T cell population. This is consistent with observations from several other populations of peripherally- and antigen-induced regulatory T cells that do not express CD25 or FoxP3 [33], [34]. However, it can not from our experiments be excluded that naturally occurring CD4^+^CD25^+^ regulatory T cells are responsible for the observed suppressive effect in epicutaneously immunized mice and this needs to be examined further. Epicutaneous immunization with autoantigens has previously been shown able to induce a suppressive/regulatory response, which prevented development of EAE [35]. The disease resistance was shown to be mediated by CD4^+^ suppressor T cells but was not dependent on CD4^+^CD25^+^ T cells. The therapeutic potential of these cells on established disease was not determined. Interestingly, other recent studies have confirmed that epicutaneous immunization may induce immune-regulatory networks with potential therapeutic advantages [36], [37]. This is an intriguing possibility, but whether the skin-induced CII-specific T cells are truly suppressive when transferred into arthritic mice has yet to be tested in our model.

Extrapolation from animal models to human disease requires great caution, but if the apparent Th2 skewing of the response following epicutaneous immunization holds true in humans, its use as therapy for chronic Th1-driven pathologies would have obvious advantages. Epicutaneous immunization is simple, painless, economical and would allow for (repetitive) self-administration. Further studies elucidating the mechanisms behind skin-induced systemic immune responses and experiments in humans should now be instigated.

In conclusion, we have shown that epicutaneous immunization can successfully control a chronic inflammatory autoimmune process, highlighting its potential future use in patients suffering from RA or other autoimmune disorders.

## Materials and Methods

### Mice

Heterozygous TCR-β transgenic SWR/J mice were extensively backcrossed with DBA/1 mice to derive the chronic collagen-induced arthritis model, as described previously [12], [38]. Mice were typed for TCR-β Tg expression by FACS and the Tg positive mice selected for experiments. Over 96% of peripheral blood T cells in Tg mice were Vβ12^+^. Male mice aged 6–8 weeks were used in this study in accordance with Home Office regulations under the Animals (Scientific Procedures) Act 1986.

### Antigens

Bovine collagen type II (CII) was purified and prepared as described [39]. In brief, for immunization, the CII was solubilized by stirring overnight at 4°C in 0.1 M acetic acid. For *in vitro* stimulation, CII was solubilized in 0.05 mM Tris-HCL and 0.2 M NaCl at pH 7.4.

### Induction and assessment of arthritis

Male DBA/1-TCR-β Tg mice were immunised with 200 µg CII emulsified in CFA by intradermal injection at the base of the tail. Mice were monitored daily and analysed every second day after the onset of clinical disease. The number of affected joints, clinical severity score and hind paw swelling were recorded by two investigators ‘blinded’ to mouse identity for the duration of each experiment. The clinical severity of arthritis was graded as follows: 0 = normal, 1 = slight swelling and/or erythema, 2 = pronounced edematous swelling, 3 = pronounced edematous swelling plus joint rigidity, 4 = laxity. Each limb was graded, allowing a maximum score of 16 for each animal. Swelling of hind paws was measured using a microcaliper (Mitutoyo, Siwa, Japan).

### Epicutaneous immunization

Mice were epicutaneously immunized with CII either 3 weeks prior or 1 week post induction of arthritis. CII used for this immunization was solubilized in acetic acid but just prior to application pH was raised to 5.2 with TRIS-HCl. For epicutaneous immunization, the stratum corneum was removed from both sides of the earlobe by gentle application and removal of cellophane tape (Scotch^TM^ (3M, Cergy-Pontoise Cedex, France)) as described [1]. Twenty-four hours later, 50 µg CII were applied to both sides of the earlobe. The application of CII to the skin was repeated on the next two consecutive days. Control animals had acetic acid/TRIS-HCl without CII applied to stripped skin in an identical manner. Two weeks after the initial epicutaneous immunization a boost was given by a single application of 50 µg CII on *de novo* stripped ear skin.

### Histological evaluation

Hind paws were removed post mortem and fixed in 10% (weight/volume) buffered formalin and decalcified in 5% EDTA. The paws were subsequently embedded in paraffin, sectioned and stained with haematoxylin and eosin (H&E) for microscopic evaluation. Histological sections were scored for severity of pathological changes by two investigators ‘blinded’ to sample identity. The severity of arthritis was scored based on the following criteria: 0 = normal, 1 = minimal synovitis and cartilage loss, no bone erosion, 2 = synovitis and erosion present but limited to discrete foci and joint architecture intact, 3 = moderate synovitis with pannus formation and erosion of cartilage and bone, 4 = severe inflammation with extensive erosion of bone and cartilage and loss of joint architecture.

### T cell proliferation and co-cultures

Spleen cell suspensions were obtained from every mouse by mechanical disaggregation and lysis of red blood cells. Splenocytes were cultured at 3×10^5^ cells in 96-well flat-bottom plates in a total volume of 200 µl RPMI 1640 medium supplemented with 10% FCS, 50 µM 2-mercaptoethanol and 5 µg/ml gentamycin. CII was added at concentrations of 50 µg/ml or 10 µg/ml. Control responses to an irrelevant antigen (OVA) at 50 µg/ml, ConA at 1 µg/ml or media alone were also determined. Cultures were incubated at 37°C for 90 h and pulsed with 1 µCi of [^3^H]-thymidine (Amersham Pharmacia, Little Chalfont, UK) for the last 16 h. Cells were harvested and thymidine incorporation determined by liquid scintillation counting on a MicroBeta (Wallac, Turku, Finland).

To determine whether cells from epicutaneously treated mice could suppress CII-specific proliferation of control cells, splenocytes from control and treated mice were co-cultured by varying the ratio between the cells in culture, while keeping the overall cell density constant at 3×10^5^ cells/well. Splenocytes from control and treated mice were cultured in ratios of 10∶0, 10∶1, 10∶5, 1∶1, 5∶10, 1∶10 and 0∶10. The cultures were stimulated with 50 µg or 10 µg CII/ml, 50 µg OVA/ml, 1 µg ConA/ml or left unstimulated for 90 h at 37°C. Cells were then pulsed with [^3^H]-thymidine, harvested and counted as described above.

### Flow cytometry

Freshly isolated splenocytes from all mice were stained for CD4 and CD25 using antibodies from PharMingen (San Diego, CA, USA) (clone GK1.5 and clone 7D4 respectively). Cells were incubated with a saturating concentration of the two antibodies or isotype controls for 30 min at 4°C in the dark. The cells were then thoroughly washed and fixed in 1% paraformaldehyde. Splenocytes from all mice were also stained for CD4^+^CD25^+^ cells following 90 h stimulation with 50 µg CII/ml in an identical manner. All cells were analyzed for two-colour cytometry staining on a FACScalibur instrument (Becton Dickinson, Mountain View, CA) and data analysis was performed with CellQuest software (Becton Dickinson). Viable lymphocytes were gated based on their forward/side scatter profile, after which CD4^+^CD25^+^ cells were gated based on expression on CD4 and CD25.

### Cytokine production

3×10^6^ splenocytes were cultured in 1 ml RPMI 1640 medium supplemented with 10% FCS, 50 µM 2-mercaptoethanol and 5 µg/ml gentamycin in 24-well tissue culture plates (Nunc, Roskilde, Denmark). Cells were stimulated with 50 µg CII/ml, 50 µg OVA/ml or left unstimulated for 90 h at 37°C. Culture supernatants were collected and subsequently assayed by ELISA for IFN-γ, IL-4, IL-10 and TGF-β using antibodies from PharMingen, according to the manufacturer's protocol. Recombinant mouse IFN-γ, IL-4, and IL-10 and human TGF-β from PharMingen were used as standards. The detection limit of the assays was 3 pg/ml for IL-4 and 40 pg/ml for IFN-γ, IL-10 and TGF-β. Supernatants were assayed for IL-13 using mouse IL-13 Quantikine ELISA kit from R&D Systems (Minneapolis, MN, USA). Detection limit for IL-13 was 8 pg/ml.

### Antibody responses

At the end of each experiment, mice were bled by cardiac puncture and sera prepared for CII-specific antibody determinations. For IgG, IgG1 and IgG2a antibodies, 96-well Maxisorb plates (Nunc) were coated with CII at 100 µg/ml in carbonate-bicarbonate buffer at 4°C over night. Optimally diluted sera (100 µl in PBS) were added and the plates incubated at 37°C for 90 min. After washing, alkaline phosphatase conjugated polyclonal goat anti-mouse IgG Fc (Sigma, Gillingham, UK), rat monoclonal antibody to mouse IgG1 (Zymed, San Francisco, CA, USA) or rat monoclonal antibody to IgG2a (PharMingen) were added for 1 h at 37°C. The alkaline phosphatase substrate pNPP (Sigma) was then added and absorbance measured at 405 nm. Total IgE was measured by an IgE capture method. Sera to be tested were added to Maxisorb microtiter plate wells coated with rat monoclonal anti-mouse IgE (PharMingen) at 1 µg/ml and incubated at 4°C overnight. Biotin conjugated monoclonal rat anti-mouse IgE (PharMingen) was then added at 1 µg/ml and incubated for 2 h at 37°C. After washing, alkaline phosphatase streptavidin (PharMingen) was added for 1 h followed by pNPP substrate.

### Statistical evaluation

The statistical significance of difference in clinical severity between experimental groups over the entire length of the experiment was determined by a 2 way ANOVA. Two-tailed Student's t-test for unpaired data was used for the other experiments. Differences were regarded as significant when p<0.05.
